# ARRIVE has not ARRIVEd: Support for the ARRIVE (Animal Research: Reporting of *in vivo* Experiments) guidelines does not improve the reporting quality of papers in animal welfare, analgesia or anesthesia

**DOI:** 10.1371/journal.pone.0197882

**Published:** 2018-05-24

**Authors:** Vivian Leung, Frédérik Rousseau-Blass, Guy Beauchamp, Daniel S. J. Pang

**Affiliations:** Faculty of Veterinary Medicine, Université de Montréal, Saint-Hyacinthe, Québec, Canada; Harvard University Faculty of Arts and Sciences, UNITED STATES

## Abstract

Poor research reporting is a major contributing factor to low study reproducibility, financial and animal waste. The ARRIVE (Animal Research: Reporting of *In Vivo* Experiments) guidelines were developed to improve reporting quality and many journals support these guidelines. The influence of this support is unknown. We hypothesized that papers published in journals supporting the ARRIVE guidelines would show improved reporting compared with those in non-supporting journals. In a retrospective, observational cohort study, papers from 5 ARRIVE supporting (SUPP) and 2 non-supporting (nonSUPP) journals, published before (2009) and 5 years after (2015) the ARRIVE guidelines, were selected. Adherence to the ARRIVE checklist of 20 items was independently evaluated by two reviewers and items assessed as fully, partially or not reported. Mean percentages of items reported were compared between journal types and years with an unequal variance t-test. Individual items and sub-items were compared with a chi-square test. From an initial cohort of 956, 236 papers were included: 120 from 2009 (SUPP; n = 52, nonSUPP; n = 68), 116 from 2015 (SUPP; n = 61, nonSUPP; n = 55). The percentage of fully reported items was similar between journal types in 2009 (SUPP: 55.3 ± 11.5% [SD]; nonSUPP: 51.8 ± 9.0%; p = 0.07, 95% CI of mean difference -0.3–7.3%) and 2015 (SUPP: 60.5 ± 11.2%; nonSUPP; 60.2 ± 10.0%; p = 0.89, 95%CI -3.6–4.2%). The small increase in fully reported items between years was similar for both journal types (p = 0.09, 95% CI -0.5–4.3%). No paper fully reported 100% of items on the ARRIVE checklist and measures associated with bias were poorly reported. These results suggest that journal support for the ARRIVE guidelines has not resulted in a meaningful improvement in reporting quality, contributing to ongoing waste in animal research.

## Introduction

Accurate and complete reporting of animal experiments is central to supporting valid, reproducible research and to allow readers to critically evaluate published work. Poor or absent reporting is associated with deficiencies in experimental design that introduce bias and exaggerated effect sizes in to the literature [1, 2]. As a result, irreproducible animal research has significant ethical and financial costs [3]. The use of animals in poorly designed studies and in efforts to reproduce such studies represents a failure to uphold the 3Rs (refine, reduce, replace) of animal research [4]. Incomplete reporting of research contributes to a waste of funding, with a conservative estimate for preclinical research, of US$28 billion annually [3].

To address low standards of reporting, the ARRIVE (Animals in Research: Reporting *In Vivo* Experiments) guidelines for reporting were published in 2010 [5, 6]. The ARRIVE guidelines are summarized by a 20 item checklist that includes reporting of measures associated with bias (randomization, blinding, sample size calculation, data handling) [7, 8]. Over 1000 journals have responded to publication of the guidelines by linking to it on their websites and in their instructions to authors [9]. The effect of these endorsements is unknown. For the majority of existing health research guidelines, the impact of journal support for other reporting guidelines on guideline adherence in published papers is unclear [10]. The impact of the CONSORT guidelines for the reporting of randomised controlled trials have been evaluated more than other reporting guidelines, and current evidence suggests that though reporting of some items has improved, overall standards of reporting remain low [11].

To our knowledge, there have been no studies comparing reporting standards between journals classified as ARRIVE guideline supporters and non-supporters. Furthermore, no studies examining adherence to the ARRIVE guidelines have been conducted in the veterinary literature. We hypothesized that papers published in supporting journals would have greater adherence to the guidelines, and therefore higher reporting standards, than those published in non-supporting journals. Additionally, we hypothesized that papers published in supporting journals would show a greater improvement in reporting standards since the guidelines became available. To test these hypotheses the related subjects of anesthetic and analgesic efficacy and animal welfare were selected for study.

## Methods

### Journal and paper selection

Journals were categorized as ARRIVE supporters (SUPP) or non-supporters (nonSUPP) based on whether the ARRIVE guidelines were mentioned in their instructions to authors when beginning the study (November 2016). Editorial offices of SUPP journals confirmed by email that the ARRIVE guidelines were included in the instructions to authors before December 2014. Papers were selected from a selection of journals from these two categories (SUPP and nonSUPP) from two years: 2009 (pre-ARRIVE) and 2015 (post-ARRIVE). SUPP journals were: Journal of the American Association for Laboratory Animal Science, Comparative Medicine, Animal Welfare, Laboratory Animals and Alternatives to Animal Experimentation. NonSUPP journals were: Applied Animal Behaviour Science and Experimental Animals. Journals were selected based on an initial search for those publishing papers on the predetermined subjects of interest (welfare, analgesic and anesthetic efficacy). Additionally, none of the selected journals had previously been included in a study assessing adherence to the ARRIVE guidelines.

An initial screening of all papers was performed by a single author (VL) by manual search of tables of contents, using titles, abstracts and keywords to identify relevant papers. Papers were selected based on subject and study type. A second screening was performed by two authors (VL and FRB) during the full text evaluation of the selected papers. Anesthesia or analgesia papers described studies assessing the efficacy of anesthetics or analgesics as a primary objective. Animal welfare papers described studies where the objective was to improve the well-being of animals used in research. Only prospective *in vivo* studies were included. Case studies were excluded.

### Evaluation

Evaluation of adherence to the ARRIVE guidelines was performed independently by two authors (VL and FRB). The ARRIVE checklist [6] of 20 items and 46 associated sub-items was operationalized and used as the basis for evaluation (Table 1). Descriptors were developed by consensus to promote consistency during evaluation (Table 1). Items without associated sub-items were categorized as either not reported, partially reported or fully reported. Items with sub-items were categorized as not reported if no sub-items were reported, partially reported if only some sub-items were reported and fully reported if all sub-items were reported. For example, for Item 6 (Study design, Table 1), the item would only be classified as fully reported if all sub-items (6a-d) were reported, otherwise it would be classified as partially (3 or fewer sub-items reported) or not reported (none of the 4 sub-items reported).

**Table 1:** The ARRIVE guidelines checklist: Operationalized items and sub-items to facilitate assessment of reporting [6].

Item/sub-item	ARRIVE items and sub-items	Possible categories	Descriptor
1	**Title**	**not reported; partially reported; fully reported**	Accurate and concise description of article content
2	**Abstract**	**not reported; partially reported; fully reported**	Accurate summary of background, research objectives, species or strain of animal used, key methods, principle findings, and conclusions
	Introduction		
3	**Background**	**depends on sub-items**	**-**
3a	Motivation for and context of study	not reported; reported	Sufficient scientific background (with references) on motivation and context of study, with explanation of experimental approach and rationale
3b	Animal species and models justified	not reported; reported	Explain how and why animal species and models were chosen
4	**Objectives**	**not reported; partially reported; fully reported**	Objectives or hypotheses of study are clearly described
	Methods		
5	**Ethical Statement**	**not reported; fully reported**	Statement to indicate ethical review permissions, relevant licenses and national or institutional guidelines for care and use of animals
6	**Study design**	**depends on sub-items**	**-**
6a	Number of groups	not reported; reported; N/A	Number of experimental and control groups clearly stated; N/A if single group study
6b	Randomization	not reported; reported; N/A	Statement that randomization was used or justification for no randomization; N/A if single group study
6c	Blinding	not reported; reported; N/A	Statement that blinding was used or justification for no blinding; N/A if single group study. Classified as “reported” if blinding was mentioned for any step (e.g. blinding to allocation, blinding to outcome assessment, treatment administration etc.).
6d	Experimental unit	not reported; reported	Reader is able to understand if comparisons were between a single animal or a group of animals
7	**Experimental procedures**	**depends on sub-items**	**-**
7a	How	not reported; reported	Description of experiment performed and details of specialised equipment used can be replicated with the information present
7b	When	not reported; reported; N/A	Statement of when during the day the procedures took place and when according to the experimental timeline; N/A if paper was assessing continuous assessment or if light cycle unlikely to affect assessment (e.g. lameness)
7c	Where	not reported; reported	Some indication of where each procedure took place
7d	Why	not reported; reported	Rationale for why chosen experimental procedures were performed
7e	Drugs used	not reported; reported	Statement of the name, dose, route, and frequency of the analgesics or anesthetics used; N/A if procedures can be obviously performed without analgesic or anesthetics
8	**Experimental animals**	**depends on sub-items**	**-**
8a	Species	not reported; reported	Statement of species used
8b	Strain	not reported; reported	Statement of strain used
8c	Sex	not reported; reported	Statement of sex used
8d	Developmental stage	not reported; reported	Statement of age of animals used
8e	Weight	not reported; reported; N/A	Statement of the animals’ weight; N/A for zoo animals
8f	Source	not reported; reported; N/A	Statement of animals’ source; N/A for zoo animals
8g	Health/immune status	not reported; reported	Statement of animals’ heath (i.e. screening of tested animals or sentinel animals for lab animals) or general statement that animals were healthy for farm, companion, and zoo animals
9	**Housing and husbandry**	**depends on sub-items**	**-**
9a	Type of cage/housing	not reported; reported; N/A	Statement of cage dimensions and product source for lab animals and a general description for companion and zoo animals; N/A if paper was on animals being process for slaughter (e.g. study at abattoir)
9b	Bedding material	not reported; reported; N/A	Statement of bedding type and source for lab animals and a general description for non-lab animals; N/A for fish species or animals being processed for slaughter
9c	Type of facility	not reported; reported; N/A	Statement of facility type and a general description for non-lab animal; N/A if paper was on animals being process for slaughter
9d	Number of cage companions	not reported; reported; N/A	Statement of number of animals housed together or individually; N/A if paper was on animals being processed for slaughter
9e	Light/dark cycle	not reported; reported; N/A	Statement of time lights were on/off for lab animals; information of place of facility and time of experiment is accepted as an alternative for farm and zoo animals*; N/A if paper was on animals being process for slaughter
9f	Temperature	not reported; reported; N/A	Statement of temperature animals were housed in; information of place of facility and time of experiment is acceptable as an alternative for farm and zoo animals*; N/A if paper was on animals being process for slaughter
9g	Type of food	not reported; reported; N/A	Statement of food type and sources for lab animals; general description (e.g. hay for cattle) acceptable for non-lab animals; N/A if paper was on animals being process for slaughter
9h	Water access	not reported; reported; N/A	Statement that water was provided; N/A for fish species or animals being processed for slaughter
9i	Environmental enrichment	not reported; reported; N/A	Statement that a form of enrichment was provided; N/A if paper was on animals being processed for slaughter
9j	Humidity	not reported; reported; N/A	Statement of humidity for lab animals; information of place and time of experiments is acceptable as an alternative for farm and zoo animals*; N/A for fish species or animals being processed for slaughter
9k	Welfare assessment	not reported; reported; N/A	Statement that a form of welfare assessment was in place; point was awarded by default if the paper was a welfare paper; N/A if the intervention performed was not for the benefit of the animals involved
9l	Welfare interventions	not reported; reported; N/A	Statement of what type of welfare intervention prepared; intervention must be in response to animals’ well-being and not from an outcome of the experiment e.g. Eye issues from eye procedure vs. Weight loss; N/A if no adverse event is expected (i.e. animal assessed after death)
9m	Time of welfare assessment or intervention	not reported; reported; N/A	Statement of when welfare assessment or intervention occurred; N/A if no adverse event expected (e.g. study was assessing a new enrichment)
10	**Sample size**	**depends on sub-items**	**-**
10a	Total number of animals used	not reported; reported	Statement specifying in absolute numbers of the total number of animals used in each experiment and treatment groups
10b	Sample size calculation	not reported; reported; N/A	Statement that sample size calculation was performed; N/A if pilot study
10c	Number of independent replications**	reported; N/A	Statement of the number of independent replications performed
11	**Allocating animals**	**depends on sub-items**	**-**
11a	Allocation method	not reported; reported; N/A	Statement of how animals were allocated to groups, including randomization or matching if done; N/A if single treatment group
11b	Treatment and assessment of animals	not reported; reported	Describe the order in which the animals in the different experimental groups were treated and assessed
12	**Experimental outcomes**	**not reported; partially reported; fully reported**	Define the primary and secondary experimental outcomes assessed
13	**Statistical methods**	**depends on sub-items**	**-**
13a	Details of statistical methods used	not reported; reported	Statistical tests performed for each analysis was clear
13b	Specify unit of analysis	not reported; reported	Unit of analysis was clear for each data set
13c	Assess normality	not reported; reported	Statement that assessment of normality was performed
	Results		
14	**Baseline data**	**not reported; fully reported**	Statement to report relevant characteristics and health status of animals were collected
15	**Numbers analysed**	**depends on sub-items**	**-**
15a	Animals included	not reported; reported	Statement of the number of animals included/excluded in absolute numbers
15b	Reasons for animal exclusion	not reported; reported; N/A	Statement detailing why animals were excluded; N/A if no animals excluded
16	**Outcomes and estimation**	**not reported; partially reported; fully reported**	Results for each analysis was clear with a measure of precision (e.g. standard error or confidence interval)
17	**Adverse events**	**depends on sub-items**	**-**
17a	Details of adverse events	not reported; reported; N/A	Reported details of adverse events that occurred or a statement to report no adverse events occurred; N/A if no adverse events expected
17b	Modifications to reduce adverse events	not reported; reported; N/A	Modifications to experimental procedures made to reduce adverse events were described; N/A if no adverse event expected
	Discussion		
18	**Interpretation/scientific implications**	**depends on sub-items**	**-**
18a	Interpretation	not reported; reported	Interpret results, taking into account study objectives and hypotheses, current theory and other relevant studies in literature
18b	Study limitations	not reported; reported	Commented on the study limitations including potential sources of bias, any limitations of the animal model and the imprecision associated with results
18c	Implications for 3Rs of animal use	not reported; reported; N/A	Described any implications of experimental methods or findings for the replacement, refinement or reduction (3Rs) of the use of animals in research; point was awarded if it was a welfare paper; N/A if assessing anatomic response to an analgesic or anesthetic (e.g. buprenorphine effects on limb volume)
19	**Generalizability/translation**	**not reported; fully reported; N/A**	Commented on whether the findings of this study are likely to translate to other species or systems, including any relevance to human biology; N/A for welfare paper unless specified in discussion
20	**Funding**	**not reported; fully reported**	List all funding sources and the role of the funder(s) in the study

Items are bolded and listed with a number. Sub-items are listed with a number and letter.

*Acceptable to report only place and time of year for 9e) light/dark cycle; 9f) temperature; 9j) humidity as this information can be inferred if animals (production and zoo types) are housed outdoors

** Number of independent replications was scored as not applicable (N/A) when not reported as this sub-item was not required for a complete study.

A sub-item was added to the original ARRIVE checklist to clarify drug use (sub-item 7e, Table 1). Where items or sub-items were considered not applicable, no score was entered. For example, a paper on zebra fish would have the sub-items bedding materials, access to water and humidity classed as not applicable.

Item and sub-item scores were compared between authors and differences resolved by consensus (with DP).

### Statistics

Each paper was assessed against the 20 items of the ARRIVE guidelines, generating percentages of fully reported items. From this, mean percentages of items were calculated for each journal type during each publication year. Following Levene’s test revealing heterogeneity of variances, an unequal variance t-test was used to compare these mean percentages between journal types (SUPP 2009 vs nonSUPP 2009; SUPP 2015 vs nonSUPP 2015) and between years (SUPP 2009 vs. SUPP 2015; nonSUPP 2009 vs. nonSUPP 2015). Correction for multiple comparisons was not applied as comparisons between identical items were viewed as independent from other items. The overall quality of item reporting was classified as well (> 80%), average (50–80%) or poor (< 50%) [12]. For each journal type, the percentages of individual items and sub-items that were fully, partially or not reported were compared between years with a chi-square test. Additionally, to provide an overall impression of reporting standards in 2015 data from both journal types were pooled.

## Results

**Fig 1:**
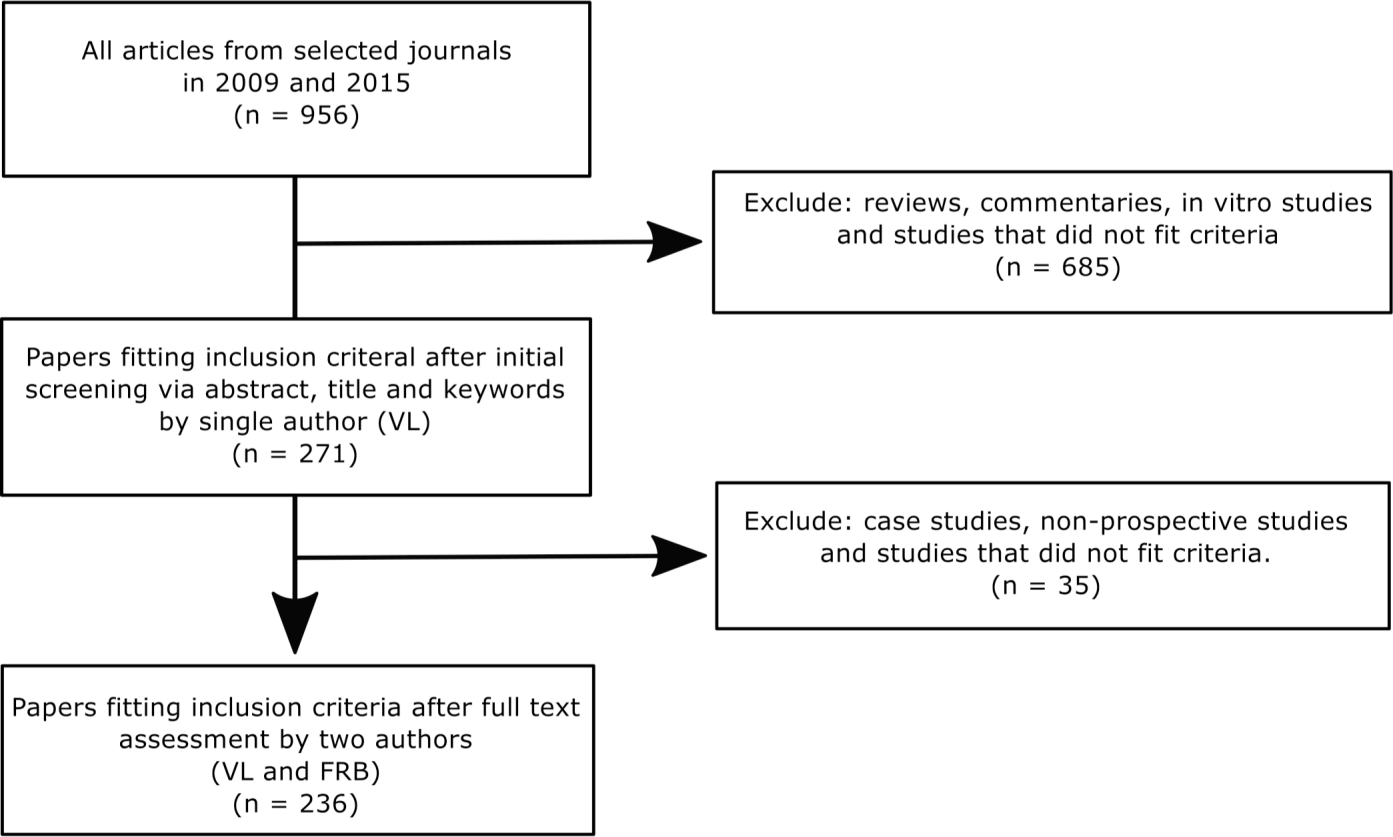
Flow diagram of paper selection process. Papers were selected from studies reporting research in anesthesia, analgesia and animal welfare from 5 veterinary journals.

After initial screening, 271 papers were identified. Thirty-five papers were excluded following full text evaluation, leaving 236 papers included in the final analysis (SUPP 2009: n = 52; SUPP 2015: n = 61; nonSUPP 2009: n = 68; nonSUPP 2015: n = 55, Fig 1). One item and one sub item (generalizability/translation (item 19), number of independent replication (sub- item 10c)) were removed before analysis as they were only applicable in a small number of papers (4/236 and 10/236, respectively). Data are available from the Harvard dataverse [13].

The percentages of fully reported items between journal types were similar in 2009 (p = 0.07) and 2015 (p = 0.89; Table 2). The percentage of fully reported items increased significantly from 2009 to 2015 for both SUPP (p = 0.02) and nonSUPP (p = 0.0001; Table 2) journals. Although both journal types showed improvements from 2009 to 2015, neither improved significantly more than the other (absolute difference in change between nonSUPP–SUPP = 3.3%, p = 0.09 [95% CI -0.5–4.3%]).

**Table 2:** Overall reporting quality in journals supporting (SUPP) and not supporting (nonSUPP) the ARRIVE guidelines for 2009 and 2015.

	2009 (%)	2015 (%)	^b^p-value [95% CI]
SUPP	55.3 ± 11.5	60.5 ± 11.2	0.02 [1.0–9.4]
Non-SUPP	51.8 ± 9.0	60.2 ± 10.0	0.0001 [5.0–11.8]
^a^p value [95% CI]	0.07 [-0.3–7.3]	0.89 [-3.6–4.2]	

Values are mean percentages of fully reported items. The numbers of papers examined were: SUPP 2009; n = 52, SUPP 2015; n = 61, nonSUPP 2009; n = 68, nonSUPP 2015; n = 55.

^a^p values of differences between journal types within the same year.

^b^p-values of differences between years for the same journal type. 95% confidence interval (95% CI) is for the mean difference.

### Items

Despite minimal improvements in overall reporting standards between 2009 and 2015, several individual items showed significant improvement in full reporting. For SUPP journals, these items were the abstract (from 69.2 to 91.8%, p = 0.003), housing and husbandry (from 3.9 to 21.3%, p = 0.01) and sample size (from 3.8 to 21.3%, p = 0.01; Table 3). For nonSUPP journals, the following items were increasingly fully reported from 2009 to 2015: ethical statement (from 36.8 to 81.8%, p < 0.0001); experimental animals (from 1.5 to 10.9%, p = 0.04) and interpretation/scientific implications (from 10.3 to 38.2%, p = 0.0004; Table 3).

**Table 3:** Papers fully reporting ARRIVE checklist items in supporting (SUPP) and non-supporting (nonSUPP) journals in 2009 and 2015.

Item		SUPP			NonSUPP		
		2009 (N = 52)	2015 (N = 61)		2009 (N = 68)	2015 (N = 55)	
		n/N (% reported)	n/N (% reported)	p-value	n/N (% reported)	n/N (% reported)	p-value
1	Title	52/52 (100)	61/61 (100)	1	68/68 (100)	55/55 (100)	1
2	Abstract	36/52 (69.2)	56/61 (91.8)	**0.003**	45/68 (66.2)	44/55 (80.0)	0.11
3	Background	52/52 (100)	60/61 (98.4)	1	68/68 (100)	55/55 (100)	1
4	Objectives	47/52 (90.2)	60/61 (98.4)	0.09	68/68 (100)	55/55 (100)	1
5	Ethical statement	39/52 (75.0)	52/61 (85.2)	0.23	25/68 (36.8)	45/55 (81.8)	**<0.0001**
6	Study design	10/52 (19.2)	19/61 (31.1)	0.20	10/68 (14.7)	15/55 (27.3)	0.12
7	Experimental procedure	34/52 (65.4)	30/61 (49.2)	0.09	45/68 (66.2)	42/55 (76.4)	0.24
8	Experimental animals	8/52 (15.4)	18/61 (29.5)	0.12	1/68 (1.5)	6/55 (10.9)	**0.04**
9	Housing and husbandry	2/51 (3.9)	13/61 (21.3)	**0.01**	3/67 (4.5)	8/54 (14.8)	0.06
10	Sample size	2/52 (3.8)	13/61 (21.3)	**0.01**	1/68 (1.5)	3/55 (5.5)	0.32
11	Allocating animals	11/52 (21.2)	16/61 (26.2)	0.66	14/68 (20.6)	17/55 (30.9)	0.22
12	Experimental outcomes	52/52 (100)	61/61 (100)	1	66/67 (98.5)	55/55 (100)	1
13	Statistical methods	23/52 (44.2)	29/61 (47.5)	0.85	38/68 (55.9)	32/55 (58.2)	0.86
14	Baseline data	24/41 (58.5)	27/50 (54.0)	0.68	20/30 (66.7)	18/35 (51.4)	0.31
15	Numbers analysed	29/52 (55.8)	39/61 (63.9)	0.44	37/68 (54.4)	25/55 (45.5)	0.37
16	Outcomes and estimation	45/52 (86.5)	49/61 (80.3)	0.45	55/68 (80.9)	49/55 (89.1)	0.32
17	Adverse events	18/29 (62.1)	17/41 (41.5)	0.15	4/18 (22.2)	8/23 (34.8)	0.50
17a	Details of adverse events	25/29 (86.2)	25/41 (61.0)	**0.03**	8/18 (44.4)	20/24 (83.3)	**0.02**
18	Interpretation/scientific implications	15/52 (28.8)	20/61 (32.8)	0.69	7/68 (10.3)	21/55 (38.2)	**0.0004**
19	Generalisability/translation	-	-	-	-	-	-
20	Funding	29/52 (55.8)	43/61 (70.5)	0.12	48/68 (70.6)	44/55 (80)	0.30

N = total number of papers where the item was applicable. n = total number of papers reporting the item. p values are for comparisons between years for each journal type.

In SUPP journals, sample size was reported at least partially by all papers in 2009 but was not reported in 9.8% of papers in 2015 (p = 0.03, S1 Table and Table 3). In both SUPP and nonSUPP journals, items that were frequently not reported in both 2009 and 2015 were baseline data, numbers analyzed and funding.

**Fig 2:**
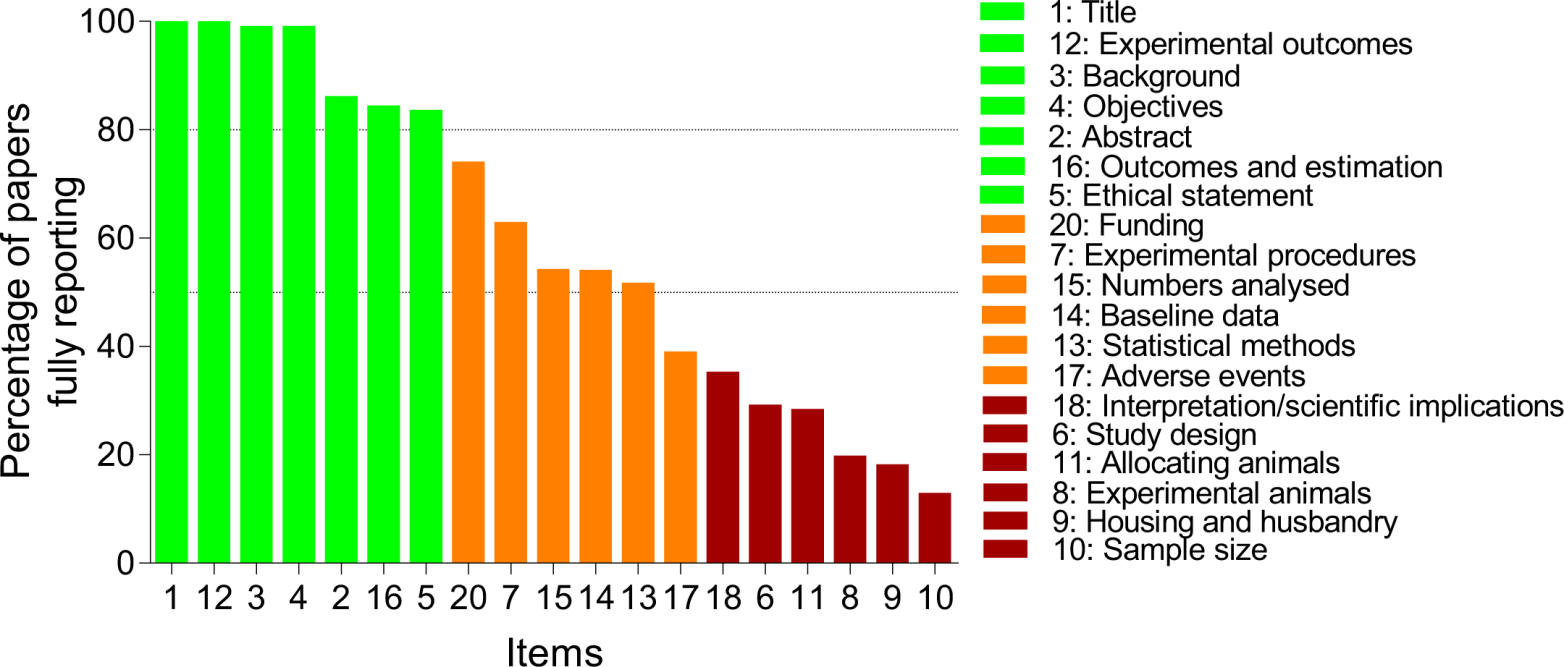
Bar graph of papers fully reporting individual items from the ARRIVE checklist. Data from papers published in 2015 were pooled from ARRIVE supporting (SUPP, n = 61 papers) and non-supporting (nonSUPP, n = 55 papers) journals. Broken horizontal lines indicate reporting quality thresholds: excellent (> 80%), average (50–80%) and poor (< 50%) [12].

Pooling the percentage of fully reported items in 2015 from both journal types revealed that items with excellent (> 80%), average (50–80%) and poor (< 50%) reporting was distributed in to thirds (Fig 2). Title, abstract, background, objectives, ethical statement, experimental outcomes, and outcomes and estimation were well reported. In contrast, ethical statement, baseline data, numbers analyzed, adverse events and funding were poorly reported.

### Sub-items

**Fig 3:**
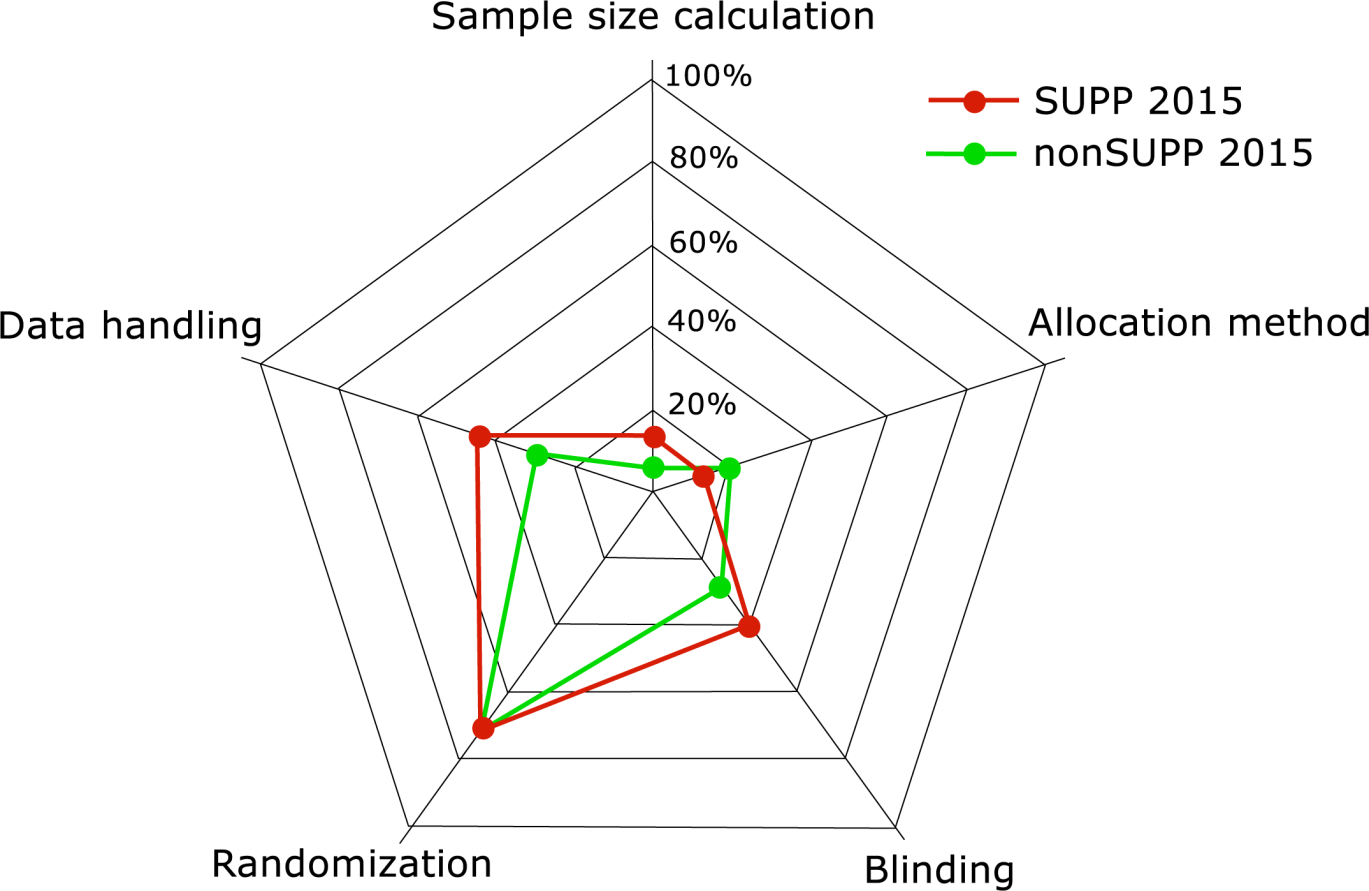
Radar plot of ARRIVE checklist sub-items associated with bias reported in ARRIVE supporting (SUPP) and non-supporting (nonSUPP) journals in 2015.

There were significant improvements in percentages of papers reporting a small number of sub-items between years for each journal type though overall levels of reporting remained low (S2 Table). Notably amongst these were sub-items associated with bias: blinding (sub-item 6c), sample size calculation (sub-item 10b), allocation method (sub-item 11a) and data handling (sub-item 15b) (Fig 3) Randomization (sub-item 6b) was alone in being reported more than 50% of the time (Fig 3).

## Discussion

Numerous studies across different research fields have shown that reporting quality has remained low since the publication of the ARRIVE guidelines [12, 14–18]. This is in spite of large scale support for the guidelines by biomedical journals and increasing awareness of the financial and ethical cost of irreproducible research [3, 5, 7, 19]. The results of our study confirm that reporting quality remains low and that journal support for the ARRIVE guidelines has not resulted in meaningful improvements in reporting standards.

### Adherence to reporting guidelines remains low despite journal support

Reporting standards in this sample of anesthesia, analgesia and animal welfare papers was low, with little indication that the ARRIVE guidelines have made an impact in improving reporting standards. These findings echo those of others [8, 15, 16]. The data presented here, published 5 years after introduction of the ARRIVE guidelines, reflect the low reporting rates identified by Kilkenny et al. (2009) [5] that served as the catalyst for creation of the guidelines. As in those findings, reporting of important indicators of study design quality (randomization, blinding, sample size calculation and data handling) remain low.

A recent study of the veterinary literature that focused on reporting of randomization in randomised controlled trials found a higher percentage pf papers (49%, n = 106) reporting the allocation method than reported here (13–20% for SUPP and nonSUPP, respectively) [20]. This difference is likely to have resulted from selecting papers self-describing as randomised clinical trials.

With the small observed increase in reported items in both SUPP and nonSUPP journals, an increased awareness of reporting standards, such as the ARRIVE guidelines, cannot be ruled out. However, these increases were limited, with no significant differences in fully reported items between journal types in 2015 and, perhaps most importantly, the reporting of key sub-items indicating bias (randomization; sub-items 6b and 11a, blinding; sub-item 6c, animals excluded; sub-item 15b and sample size calculation; sub-item 10b) remained low [7, 8]. Similar findings have been reported in surveys of experimental animal models, including acute lung injury, peri-odontology, autoimmunity and neoplasia [14–18]. Sample size justification, in particular, is consistently poorly reported, with reporting percentages ranging from 0–7% [14–18]. This is an alarming figure given the impact it has on interpretation of findings and animal use [21].

A common feature in this and other studies of ARRIVE guideline adherence has been a lack of enforcement of reporting standards. In contrast, when reporting is mandatory, important improvements have been achieved [22, 23]. Following a change in editorial policy in 2013, the Nature research journals now require that authors accompany accepted manuscripts with a completed checklist identifying inclusion of key items associated with quality of reporting and study design [24]. This checklist has numerous items in common with those of the ARRIVE guidelines. In reviewing approximately 440 papers in each of two groups (those published in the Nature publishing journals and those from other publishers, before and after checklist implementation), the positive effect of the checklist was evident in that reporting of bias criteria (randomization, blinding, sample size calculation and data handling) [7] improved significantly from 0 to 16.4% [23]. While this number remains low, the percentage of papers from other publishers reporting these items was < 1% over the same time period. In striking contrast with the findings presented here and elsewhere [14–18], introduction of the checklist was associated with a mention of sample size calculation in 58% (90/154) of papers, increasing from < 2% (3/192).

### Suggestions to improved guideline adherence

To date, a change in editorial policy accompanied by mandatory submission of a reporting checklist is the only method shown to have resulted in an increase in reporting quality [23]. This clearly indicates that enforcement is required to generate a change in behavior. As others have suggested, achieving change in a well-established process, such as peer-review, is difficult [25]. Furthermore, placing the responsibility of policing guideline adherence on reviewers is unrealistic, when they are volunteering their time, usually busy and may share the same view of an unenforced request to complete a checklist [7, 25].

Other, albeit untested, suggestions to improve reporting standards include: 1. using a template of the methods section to require completion of desired items [25], 2. standardizing reporting of common outcomes by learned societies and research communities [15, 26–29] and 3. mandating adherence to reporting standards at the stage of applying for federal authority to conduct research (in countries where this applies), perhaps in the form of study registration [30]. These suggestions, along with the checklist used by the Nature research journals, represent a shift away from the current format of the ARRIVE guidelines towards a shorter checklist. Irrespective of scope and format, it is clear reporting standards will remain low without some form of enforced adherence [15, 25]. An important consequence of enforced compliance, which must be considered when selecting a method to improve reporting, is the associated cost (time and financial resources) to publishers and authors, and striking an acceptable balance between an ideal and that which is feasible, practical and achievable.

### Limitations

Our data may have been skewed by the small number of journals in the nonSUPP group and any policies of individual journals on how compliance with the ARRIVE reporting guidelines were assessed. The choice of journals was limited due to the large number that have registered support for the ARRIVE guidelines and our choice of subject matter. While this reflects the success of the ARRIVE guidelines in being widely adopted, our data highlight that the relationship between guideline support and adherence merits investigation [15, 31]. Despite the low number of journals included, the risk of systematic journal bias is likely to be low given similar standards of reporting have been documented across a wide range of biomedical journals [12, 14–18].

### Conclusion

Journal support for the ARRIVE guidelines has not resulted in improved reporting standards, with the lowest levels of reporting associated with factors reflecting potential study bias. To achieve meaningful improvements in reporting standards, as a means to improve study reproducibility and reduce financial and animal waste, enforcement of reporting is necessary.

## Supporting information

S1 Table Papers partially reporting ARRIVE checklist items in supporting (SUPP) and non-supporting (nonSUPP) journals in 2009 and 2015. N = total number of papers where the item was applicable. n = total number of papers partially reporting the item. p values are for comparisons between years for each journal type.
(DOCX)

S2 Table Papers fully reporting ARRIVE checklist sub-items in supporting (SUPP) and non-supporting (nonSUPP) journals in 2009 and 2015. N = total number of journal articles where the sub-item was applicable; n = total number of journal articles reporting the sub-item. p values are for comparisons between years for each journal type.
(DOCX)
