# Supplementary material for: ARRIVE has not ARRIVEd: Support for the ARRIVE (Animal Research: Reporting of in vivo Experiments) guidelines does not improve the reporting quality of papers in animal welfare, analgesia or anesthesia
Source: PLoS One. 2018 May 24;13(5):e0197882. doi: 10.1371/journal.pone.0197882 (PMC5967836; doi:10.1371/journal.pone.0197882)
Supplement: S1 Table — N = total number of papers where the item was applicable. n = total number of papers partially reporting the item. p values are for comparisons between years for each journal type. (DOCX) [file pone.0197882.s001.docx]

**S1 Table. Papers partially reporting ARRIVE checklist items in supporting (SUPP) and non-supporting (nonSUPP) journals in 2009 and 2015.**

| Item | | SUPP | | | NonSUPP | | |
| --- | --- | --- | --- | --- | --- | --- | --- |
|  |  | 2009 (N = 52) | 2015 (N = 61) |  | 2009 (N = 68) | 2015 (N = 55) |  |
|  |  | n/N (% reported) | n/N (% reported) | p-value | n/N (% reported) | n/N (% reported) | p-value |
| 1 | Title | 0/52 (0) | 0/61 (0) | 1 | 0/68 (0) | 0/55 (0) | 1 |
| 2 | Abstract | 16/52 (30.8) | 5/61 (8.2) | 0.003 | 23/68 (33.8) | 11/55 (20.0) | 0.11 |
| 3 | Background | 0/52 (0) | 1/61 (1.6) | 1 | 0/68 (0) | 0/55 (0) | 1 |
| 4 | Objectives | 1/52 (2.0) | 0/61 (0) | 0.46 | 0/68 (0) | 0/55 (0) | 1 |
| 5 | Ethical statement | 0/52 (0) | 0/61 (0) | 1 | 0/68 (0) | 0/55 (0) | 1 |
| 6 | Study design | 42/52 (80.8) | 42/61 (68.9) | 0.20 | 58/68 (85.3) | 40/55 (72.7) | 0.12 |
| 7 | Experimental procedure | 28/52 (34.6) | 31/61 (50.8) | 0.09 | 23/68 (33.8) | 13/55 (23.6) | 0.24 |
| 8 | Experimental animals | 44/52 (84.6) | 43/61 (70.5) | 0.12 | 67/68 (98.5) | 49/55 (89.1) | 0.04 |
| 9 | Housing and husbandry | 49/51 (96.1) | 48/61 (78.7) | 0.01 | 64/67 (95.5) | 46/54 (85.2) | 0.06 |
| 10 | Sample size | 50/52 (96.2) | 42/61 (68.9) | 0.0002 | 67/68 (98.5) | 51/55 (92.7) | 0.17 |
| 11 | Allocation animals | 41/52 (78.8) | 45/61 (73.8) | 0.66 | 54/68 (79.4) | 38/55 (69.1) | 0.22 |
| 12 | Experimental outcomes | 0/52 (0) | 0/61 (0) | 1 | 1/67 (1.5) | 0/55 (0) | 1 |
| 13 | Statistical methods | 27/52 (51.9) | 30/61 (49.2) | 0.85 | 25/68 (36.8) | 23/55 (41.8) | 0.58 |
| 14 | Baseline data | 1/41 (2.4) | 0/50 (0) | 0.45 | 0/30 (0) | 0/35 (0) | 1 |
| 15 | Numbers analysed | 4/52 (7.7) | 2/61 (3.3) | 0.31 | 3/68 (4.4) | 2/55 (3.6) | 1 |
| 16 | Outcomes and estimation | 6/52 (11.5) | 11/61 (18.0) | 0.43 | 13/68 (19.1) | 6/55 (10.9) | 0.32 |
| 17 | Adverse events | 7/29 (24.1) | 9/41 (2.0) | 1 | 4/18 (22.2) | 10/23 (43.5) | 0.2 |
| 18 | Interpretation/scientific implications | 37/52 (71.2) | 41/61 (67.2) | 0.69 | 61/68 (87.7) | 34/55 (61.8) | 0.0004 |
| 19 | Generalisability/translation |  |  |  |  |  |  |
| 20 | Funding | 0/52 (52.0) | 0/61 (0) | 1 | 0/68 (0) | 0/55 (0) | 1 |

N = total number of papers where the item was applicable. n = total number of papers partially reporting the item. p values are for comparisons between years for each journal type.
