# Supplementary material for: ARRIVE has not ARRIVEd: Support for the ARRIVE (Animal Research: Reporting of in vivo Experiments) guidelines does not improve the reporting quality of papers in animal welfare, analgesia or anesthesia
Source: PLoS One. 2018 May 24;13(5):e0197882. doi: 10.1371/journal.pone.0197882 (PMC5967836; doi:10.1371/journal.pone.0197882)
Supplement: S2 Table — N = total number of journal articles where the sub-item was applicable; n = total number of journal articles reporting the sub-item. p values are for comparisons between years for each journal type. (DOCX) [file pone.0197882.s002.docx]

**S2 Table. Papers fully reporting ARRIVE checklist sub-items in supporting (SUPP) and non-supporting (nonSUPP) journals in 2009 and 2015.**

| Items | Sub-items | | SUPP | | | NonSUPP | | |
| --- | --- | --- | --- | --- | --- | --- | --- | --- |
|  |  |  | 2009 (N = 52) | 2015 (N = 61) |  | 2009 (N = 68) | 2015 (N = 55) |  |
|  |  |  | N (% reported) | n (% reported) | p-value | n (% reported) | n (% reported) | p-value |
| Background | 3a | Motivation and context of study | 52/52 (100) | 61/61 (100) | 1 | 68/68 (100) | 55/55 (100) | 1 |
|  | 3b | Animal species and model justification | 52/52 (100) | 60/61 (98.4) | 1 | 68/68 (100) | 55/55 (100) | 1 |
| Study design | 6a | Number of groups | 52/52 (100) | 61/61 (100) | 1 | 68/68 (100) | 55/55 (100) | 1 |
|  | 6b | Randomization | 29/46 (63.0) | 37/52 (71.2) | 0.52 | 38/67 (56.7) | 34/48 (70.8) | 0.17 |
|  | 6c | Blinding | 13/52 (25.0) | 24/60 (40.0) | 0.11 | 10/68 (14.7) | 15/53 (28.3) | 0.08 |
|  | 6d | Experimental units | 52/52 (100) | 61/61 (100) | 1 | 68/68 (100) | 55/55 (100) | 1 |
| Experimental procedure | 7a | How | 52/52 (100) | 61/61 (100) | 1 | 68/68 (100) | 55/55 (100) | 1 |
|  | 7b | When | 31/44 (70.5) | 36/60 (60.0) | 0.31 | 47/68 (69.1) | 45/54 (83.3) | 0.09 |
|  | 7c | Where | 45/52 (86.5) | 55/61 (90.2) | 0.57 | 67/68 (98.5) | 52/55 (94.5) | 0.33 |
|  | 7d | Why | 52/52 (100) | 61/61 (100) | 1 | 68/68 (100) | 55/55 (100) | 1 |
|  | 7e | Drugs used | 20/20 (100) | 29/33 (87.9) | 0.17 | 5/6 (83.3) | 9/10 (90) | 1 |
| Experimental animals | 8a | Species | 52/52 (100) | 61/61 (100) | 1 | 68/68 (100) | 55/55 (100) | 1 |
|  | 8b | Strain | 48/52 (92.3) | 61/61 (100) | 0.04 | 62/68 (91.2) | 49/54 (90.7) | 1 |
|  | 8c | Sex | 46/52 (88.5) | 60/61 (98.4) | 0.05 | 55/68 (80.9) | 50/55 (90.9) | 0.13 |
|  | 8d | Developmental stage | 38/52 (73.1) | 49/61 (80.3) | 0.38 | 49/68 (72.1) | 45/55 (81.8) | 0.29 |
|  | 8e | Weight | 28/49 (57.1) | 32/58 (55.2) | 0.85 | 26/63 (41.3) | 23/50 (46) | 0.70 |
|  | 8f | Source | 34/52 (65.4) | 52/61 (85.2) | 0.02 | 34/68 (50) | 32/55 (58.2) | 0.47 |
|  | 8g | Health/immune Status | 19/52 (36.5) | 34/61 (55.7) | 0.06 | 7/68 (10.3) | 7/55 (12.7) | 0.78 |
| Housing and husbandry | 9a | Type of cage/housing | 42/50 (84.0) | 49/55 (89.1) | 0.57 | 59/66 (89.4) | 51/53 (96.2) | 0.19 |
|  | 9b | Bedding material | 34/48 (70.8) | 36/52 (69.2) | 1 | 39/61 (63.9) | 42/51 (82.4) | 0.04 |
|  | 9c | Type of facility | 23/50 (46.0) | 33/54 (61.1) | 0.17 | 39/66 (59.1) | 33/53 (62.3) | 0.85 |
|  | 9d | Number of cage companions | 47/50 (94.0) | 51/55 (92.7) | 1 | 62/66 (93.9) | 49/53 (92.5) | 1 |
|  | 9e | Light/dark cycle | 36/50 (72.0) | 46/55 (83.6) | 0.17 | 22/66 (33.3) | 29/53 (54.7) | 0.025 |
|  | 9f | Temperature | 34/50 (68.0) | 35/55 (63.6) | 0.68 | 21/66 (31.8) | 30/53 (56.6) | 0.009 |
|  | 9g | Type of food | 45/50 (90.0) | 47/54 (87.0) | 0.76 | 63/67 (94.0) | 44/53 (83.0) | 0.076 |
|  | 9h | Water access | 42/49 (85.7) | 43/52 (82.7) | 0.79 | 42/61 (68.9) | 41/52 (78.8) | 0.29 |
|  | 9i | Environmental enrichment | 25/50 (50.0) | 29/54 (53.7) | 0.84 | 35/66 (53.0) | 23/53 (43.4) | 0.36 |
|  | 9j | Humidity | 25/48 (52.1) | 25/53 (47.2) | 0.69 | 12/61 (19.7) | 20/52 (38.5) | 0.04 |
|  | 9k | Welfare assessment | 45/51 (88.2) | 51/59 (86.4) | 1 | 66/68 (97.1) | 52/55 (94.5) | 0.66 |
|  | 9l | Welfare interventions | 12/27 (44.4) | 11/27 (40.7) | 1 | 1/7 (14.3) | 8/22 (36.4) | 0.38 |
|  | 9m | Time of welfare assessment or intervention | 43/50 (86.0) | 52/60 (86.7) | 1 | 66/68 (97.1) | 51/55 (92.7) | 0.41 |
| Sample size | 10a | Total number of animals used | 52/52 (100) | 56/61 (91.8) | 0.06 | 67/68 (98.5) | 53/55 (96.4) | 0.59 |
|  | 10b | Sample size calculation | 1/52 (1.9) | 8/59 (13.6) | 0.04 | 2/68 (2.9) | 3/55 (5.5) | 0.66 |
|  | 10c | Sample size: Number of independent replication | - | - | - | - | - | - |
| Animal allocation | 11a | Allocation method | 5/49 (10.2) | 7/61 (13.2) | 0.76 | 10/64 (15.6) | 10/50 (20) | 0.62 |
|  | 11b | Treatment and assessment of animals | 52/52 (100) | 61/61 (100) | 1 | 68/68 (100) | 55/55 (100) | 1 |
| Statistical methods | 13a | Details of statistical methods used | 50/52 (96.2) | 59/61 (96.7) | 1 | 64/68 (94.1) | 55/55 (100) | 0.13 |
|  | 13b | Specify unit of analysis | 48/52 (92.3) | 57/61 (93.4) | 1 | 63/68 (92.6) | 55/55 (100) | 0.06 |
|  | 13c | Assess normality | 27/52 (51.9) | 29/61 (47.5) | 0.71 | 38/68 (55.9) | 34/55 (61.8) | 0.58 |
| Numbers analyzed | 15a | Animals included | 31/52 (59.6) | 42/61 (68.9) | 0.33 | 40/68 (58.8) | 27/55 (49.1) | 0.36 |
|  | 15b | Reasons for animal exclusion | 13/33 (39.4) | 17/37 (45.9) | 0.63 | 18/45 (40) | 13/41 (31.7) | 0.50 |
| Adverse Events | 17a | Details of adverse events | 25/29 (86.2) | 25/41 (61.0) | 0.03 | 8/18 (44.4) | 20/24 (83.3) | 0.02 |
|  | 17b | Modifications to reduce adverse events | 8/19 (42.1) | 8/30 (26. 7) | 0.35 | 1/15 (6.7) | 5/20 (25) | 0.21 |
| Interpretation | 18a | Interpretation | 52/52 (100) | 61/61 (100) | 1 | 68/68 (100) | 55/55 (100) | 1 |
|  | 18b | Study limitations | 16/52 (30.8) | 22/61 (36.1) | 0.69 | 7/68 (10.3) | 20/55 (36.4) | 0.0008 |
|  | 18c | Implications for 3Rs of animal use | 51/51 (100) | 61/61 (100) | 1 | 68/68 (100) | 55/55 (100) | 1 |

N = total number of journal articles where the sub-item was applicable; n = total number of journal articles reporting the sub-item. p values are for comparisons between years for each journal type.
